# Clinical outcomes and safety of Diphoterine^®^ irrigation for chemical eye injury: A single-centre experience in the United Kingdom

**DOI:** 10.1177/25158414211030429

**Published:** 2021-07-16

**Authors:** Muzammil Ahmad Nahaboo Solim, Teresa Maria Lupion-Duran, Romeela Rana-Rahman, Trushar Patel, Desiree Ah-Kine, Darren S. J. Ting

**Affiliations:** Department of Ophthalmology, James Cook University Hospital, Middlesbrough, UK; Faculty of Medical Sciences, Newcastle University, Newcastle upon Tyne, UK; Department of Ophthalmology, James Cook University Hospital, Middlesbrough, UK; Department of Ophthalmology, James Cook University Hospital, Middlesbrough, UK; Department of Ophthalmology, James Cook University Hospital, Middlesbrough, UK; Department of Ophthalmology, James Cook University Hospital, Middlesbrough, UK; Academic Ophthalmology, Division of Clinical Neuroscience, School of Medicine, University of Nottingham, Nottingham NG7 2RD, UK; Department of Ophthalmology, The James Cook University Hospital, Middlesbrough, UK; Department of Ophthalmology, Queen’s Medical Centre, Nottingham, UK

**Keywords:** chemical eye burn, chemical eye injury, Diphoterine, irrigation, ocular surface

## Abstract

**Purpose:**

Diphoterine^®^ is an amphoteric irrigating solution armed with rapid pH-neutralising action. It serves as an effective first-aid treatment for managing chemical burns, including chemical eye injury (CEI). However, its use is not widely adopted in current clinical practice, primarily attributed to limited clinical evidence. This study aims to highlight the experience in using Diphoterine for managing CEI in a UK tertiary referral centre.

**Methods:**

This retrospective case series included all patients who presented with CEI and treated with Diphoterine at the James Cook University Hospital, UK, between April 2018 and February 2020.

**Results:**

Seven patients (10 eyes) were included; the mean age was 28.2 ± 17.0 years (ranged, 3–70 years) and 85.7% were male. All patients presented with an alkaline injury with a mean presenting pH of 8.7 ± 0.7 and a median (±interquartile range [IQR]) corrected-distance visual acuity (CDVA) of 0.10 ± 0.28 logMAR. Based on Roper-Hall classification, 90% and 10% of the eyes were of grade-I and -IV CEI, respectively. All eyes received normal saline/water as the first irrigation fluid and Diphoterine as second irrigation fluid. The mean pH improved slightly after first irrigation (8.4 ± 0.7; *p* = 0.13) and significantly after second irrigation (7.6 ± 0.4; *p* = 0.001). The volume of irrigation used was significantly less for Diphoterine (520 ± 193 mL) than for normal saline/water (2700 ± 2451 mL; *p* = 0.016). At final follow-up (median = 5 days), the median CDVA remained stable at 0.10 ± 0.28 logMAR (*p* = 0.60). One patient developed near-total limbal stem cell deficiency as a complication of grade-IV injury and was awaiting limbal stem cell transplantation at last follow-up.

**Conclusion:**

This study represents the first case series in the United Kingdom, reporting the use of Diphoterine in managing CEI. The rapid pH-neutralising action of Diphoterine, with less volume required, makes it an ideal initial treatment for efficiently managing adult and paediatric patients with CEI in clinics.

## Introduction

Chemical eye injury (CEI) is a common ophthalmic emergency with potential sight-threatening complications.^1^ The incidence of CEI is estimated to range from 5.6 to 50 per 100,000 people per year, with higher rates noted in the developing countries.^1–3^ In severe cases of CEI, it has been shown to cause significant economic and humanistic burdens.^4^ Visual prognosis of CEI is largely dependent on various factors, including the type of chemical agents, timeliness of receiving appropriate treatment, and severity of the initial injury.^1^ Among all, timely irrigation of the eye to remove the chemical irritants is recognised as the most important intervention in managing CEI. It has been shown to reduce the CEI severity and improve final visual outcome.^1^ That said, there is much debate regarding the type of irrigation fluid to be used.^5^

Diphoterine® is a sterile solution with amphoteric, polyvalent, and chelating properties and is licenced in Europe as a class-II medical device for use for chemical injuries involving the skin and eye.^6,7^ Compared to saline which works by diluting the chemicals at the site of injury, Diphoterine has active binding sites for both acidic and alkali agents,^8^ rendering it a more effective treatment for CEI. Despite its ability to rapidly neutralise the pH and reduce tissue necrosis,^6^ Diphoterine has not been widely adopted in the management of CEI in many countries, including the United Kingdom, primarily attributed to the limited clinical evidence available in the literature.^6,9–11^ In view of the paucity of literature, our study aims to report the clinical outcomes and safety of Diphoterine in managing CEI in the United Kingdom.

## Materials and methods

This was a retrospective, interventional case series examining the effectiveness and tolerability of Diphoterine as an irrigation agent for CEI. We included all patients who presented with CEI and treated with Diphoterine at the James Cook University Hospital, UK, between April 2018 and February 2020. Relevant data, including patients’ demographic factors, mode of injury, chemical agents, severity of injury (based on Roper-Hall grading; Table 1),^12^ irrigation fluid used, and visual outcome, were obtained from the medical case notes.

**Table 1.** Roper-Hall classification of chemical eye injury.

Grade	Cornea	Limbal ischaemia	Prognosis
I	Corneal epithelial damage	None	Good
II	Corneal haze, iris details visible	<33%	Good
III	Total epithelial loss, stromal haze, iris details obscured	33–50%	Guarded
IV	Cornea opaque, iris and pupil obscured	>50%	Poor

Based on our local protocol, all patients presenting with CEI underwent surface pH measurement and eye irrigation immediately upon arrival at the emergency department. After the first irrigation, the pH was re-tested after 10 to 15 mins. Subsequent irrigation and pH testing were repeated until the pH was normalised. All patients were examined by the ophthalmologists using slit-lamp biomicroscopy and fluorescein staining, particularly assessing for any corneal and conjunctival injuries (e.g. corneal haze, corneal melt, epithelial defect, and limbal ischaemia), eyelid injury, anterior chamber inflammation, intraocular pressure, and lens damage. Discharge criteria included complete resolution of epithelial defect and inflammation at the ocular surface and/or normalisation of corrected-distance visual acuity (CDVA) to baseline.

For statistical analysis, CDVA was converted from Snellen vision to logMAR vision, presented in median ± interquartile range (IQR). Paired student *T*-test was performed to analyse the mean difference between two groups. Ethical approval was waived as this study was considered a clinical service evaluation study by the clinical governance team of the South Tees Hospitals NHS Foundation Trust, Middlesbrough, UK. All treatment provided in this study formed part of the standard practice of managing CEI in our unit. Written informed consent was obtained from the patients for publication of the medical data and images.

## Results

A total of 10 eyes (7 patients) were included; the mean age was 28.2 ± 17.0 years (ranged 3–70 years) with an 85.7% male preponderance (Tables 2 and 3). The most common mode of injury was occupational accidental injury (4 patients; *n* = 5 eyes, 50%), followed by domestic accidental injury (2 patients; *n* = 3 eyes, 30%), and assault-related injury (1 patient; *n* = 2 eyes, 20%). None of the patients reported the use of eye protection at the time of injury. All (100%) patients presented with an alkaline injury with a mean presenting pH of 8.7 ± 0.7 and a median CDVA of 0.10 ± 0.28 logMAR. Based on Roper-Hall classification, 9 (90%) and 1 (10%) of the eyes were of grade-I and -IV CEI, respectively. All the patients received normal saline/water as the first irrigation fluid and followed by Diphoterine as the second irrigation fluid. The mean pH improved slightly after the first irrigation (mean pH of 8.4 ± 0.7; *p* = 0.13) and significantly after the second irrigation (mean pH of 7.6 ± 0.4; *p* = 0.001). The volume of irrigation used was significantly less for Diphoterine (520 ± 193 mL) than normal saline/water (2700 ± 2451 mL; *p* = 0.016). No Diphoterine-related allergic or toxic reaction was observed in our study. At final follow-up, the median CDVA remained stable at 0.10 ± 0.28 logMAR, with no adverse event being noted in the majority (*n* = 9, 90%) of eyes. One eye developed near-total limbal stem cell deficiency (LSCD) following a grade-IV CEI (see below ‘*Patient 3*’).

**Table 2.** Summary of the baseline characteristics, types, and volumes of irrigation fluid used and pH changes of all patients presented with chemical eye injury.

Parameters	Results	*p* value
Patients age (years)	28.2 ± 17.0 (range 3–70)	
Males	6 (85.7%)	
Mode of injury		
Accidental (Domestic)	2 patients (3 eyes; 30%)	
Accidental (Occupational)	4 patients (5 eyes; 50%)	
Assault	1 patient (2 eyes; 20%)	
Chemical substances		
Alkaline	7 patients (10 eyes; 100.0%)	
Acid	0 (0%)	
Severity (Roper-Hall classification)		
Grade I	6 patients (9 eyes, 90%)	
Grade IV	1 patient (1 eye, 10%)	
First irrigation fluid		
Normal saline	5 patients (6 eyes; 60%)	
Water (at home)	2 patients (4 eyes; 40%)	
Second irrigation fluid		
Diphoterine	7 patients (10 eyes; 100%)	
pH		
At presentation	8.7 ± 0.7	–
After 1st irrigation	8.4 ± 0.7	0.13^a^
After 2nd irrigation	7.8 ± 0.6	0.001^b^
Volume (ml) of irrigation fluid		0.016
1st irrigation fluid (Normal saline or water)	2700 ± 2451	
2nd irrigation fluid (Diphoterine)	520 ± 193	

a pH after 1st irrigation compared to pH at presentation.

b pH after 2nd irrigation compared to pH at presentation.

**Table 3.** Details of the patients presenting with chemical eye injury that received eye irrigation with Diphoterine at James Cook University Hospital, UK.

Case	Age	Gender	Nature of injury	Severity (Roper-Hall)	Eye	1st irrigation and volume (ml)	2nd irrigation and volume (ml)	Initial pH	pH after 1st irrigation	pH after 2nd irrigation	BCVA at presentation (logMAR)	BCVA at discharge (logMAR)	Adverse events	Duration of follow-up
1	30	Male	Occupational	I	Right	Normal saline (9000)	Diphoterine (1000)	9	9	7	0.0	–0.20	None	2 days
2	3	Female	Domestic	I	Right	Normal saline (2000)	Diphoterine (500)	9	8	7	0.0	0	None	3 days
3	37	Male	Occupational	IV	Right	Normal saline (4000)	Diphoterine (200)	8	8	7.5	0.30	0.80	LSCD from injury	15 months
4	23	Male	Assault	I	Right	Water (1000)	Diphoterine (500)	8	8	8	0.20	0.20	None	7 days
4	23	Male	Assault	I	Left	Water (1000)	Diphoterine (500)	9	9	8	0.20	0.20	None	7 days
5	25	Male	Occupational	I	Right	Normal saline (3000)	Diphoterine (500)	9	9	8	0.30	0.30	None	5 days
5	25	Male	Occupational	I	Left	Normal saline (2000)	Diphoterine (500)	9	7	7	0.30	0.30	None	5 days
6	23	Male	Domestic	I	Right	Normal saline (1000)	Diphoterine (500)	8	8	7.5	0.0	0.0	None	2 days
6	23	Male	Domestic	I	Left	Normal saline (1000)	Diphoterine (500)	8	8	7.5	0.0	0.0	None	2 days
7	70	Male	Occupational	I	Right	Normal saline (3000)	Diphoterine (500)	10	9.5	8	0.0	0.0	None	33 days

BCVA, best-corrected-distance-visual-acuity; LSCD, limbal stem cell deficiency.

### Representative case studies

#### Patient 1 (adult patient, grade-1 CEI, good outcome)

A 30-year-old male patient presented to the eye emergency department with a right grade-1 occupational CEI. His presenting vision was 0.0 logMAR in the right eye. The presenting pH was 9 and had remained at the same level following 9 L of normal saline. In view of the persistently high pH, 1000 mL (2 × 500 mL cannisters) of Diphoterine was administered, which successfully neutralised the pH to 7. Examination revealed a small inferior conjunctival burn with no corneal haze or limbal ischaemia. The patient was treated with preservative-free chloramphenicol and prednisolone eye drops for 1 week. At 2-day follow-up, the conjunctival defect had completely healed, and the patient was discharged with a right CDVA of −0.2 logMAR.

#### Patient 2 (paediatric patient, grade-1 CEI, good outcome)

A 3-year-old girl presented to the eye emergency department with a right grade-I CEI following a domestic accident. The presenting pH was 9 and was reduced to 8 following 2 L of normal saline irrigation. Due to inadequate control of the pH, the eye was further irrigated with 500 mL of Diphoterine, which effectively normalised the pH to 7. On examination, the patient had mild conjunctival injection of the eye, with a localised small area of conjunctival fluorescein staining from 4 to 6 o’clock. There was no corneal staining or haze, or limbal ischaemia. The patient was started on chloramphenicol eye drops and was discharged on Day 3 with no complications with a right CDVA of 0.0 logMAR.

#### Patient 3 (adult patient, grade-4 CEI, guarded outcome)

**Figure 1.**
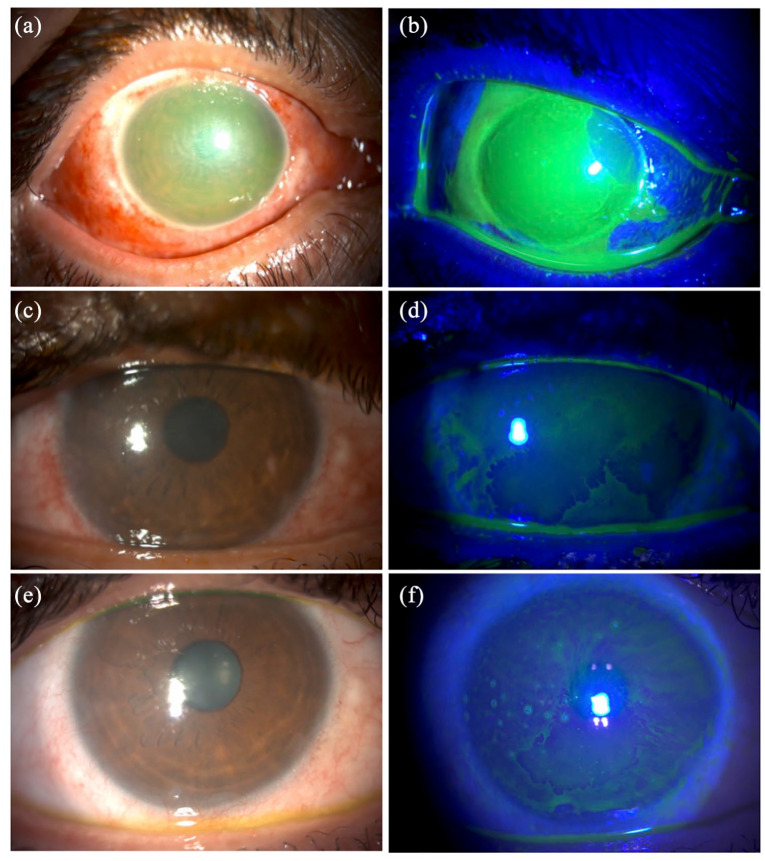
A patient presented with right grade-IV occupational chemical eye injury (CEI). (a and b) Slit-lamp photographs demonstrating a near-total limbal ischaemia spanning 10 clock-hours (from 3 to 1 o’clock), evidence by the limbal staining and whitening. The cornea appears to be hazy and oedematous, obscuring the iris details. (c and d) At 6 months post-injury, slit-lamp photographs demonstrating a right partial limbal stem cell deficiency (LSCD), evidenced by the stippled fluorescein staining that affected the visual axis. The vision was 0.80 logMAR. A small central 3 mm epitheliectomy was performed and improved the vision to 0.20 logMAR. (e and f) At 15 months post-injury, slit-lamp photographs demonstrating right complete LSCD affecting the visual axis, despite superficial epitheliectomy. Vision remained at 0.80 logMAR.

A 37-year-old male presented to the eye emergency department with a right grade-IV occupational CEI, approximately 30 mins following the injury. The patient received 4 L of normal saline, following which the pH remained at 8. This was followed by 200 mL of Diphoterine, which reduced the pH to 7.5. The presenting right CDVA was 0.30 logMAR. Slit-lamp examination revealed a right injected eye, with conjunctival oedema and limbal ischaemia spanning 10 clock-hours (3 to 1 o’clock position; Figure 1(a) and (b)). The patient was treated with preservative-free topical dexamethasone, chloramphenicol, cyclopentolate, citrate, and oral vitamin C and doxycycline. The patient subsequently developed partial LSCD, evidenced by conjunctivalisation of about two-thirds of the cornea with stippled fluorescein staining. At 6-month follow-up, the patient complained of ongoing reduced vision (CDVA of 0.80 logMAR) due to LSCD affecting the visual axis (Figure 1(c) and (d). A small 3-mm central epitheliectomy was performed to remove the conjunctivalised area from the affected cornea and promote normal corneal re-epithelialisation. The right CDVA improved significantly to 0.20 logMAR at 1-week postoperative but deteriorated to 0.80 logMAR by 1-month postoperative due to recurrence of conjunctivalisation and LSCD. At last follow-up (15 months post-injury), the right vision remained at 0.80 logMAR and the patient was placed on the waiting list for simple limbal epithelial transplantation to treat his persistent LSCD (Figure 1(e) and (f)).

## Discussion

Diphoterine is a polyvalent, amphoteric, and slightly hypertonic solution that was first developed in France and is licenced in Europe as a class II medical device for treating chemical injury of skin and eyes.^6,7^ Pre-clinical studies have demonstrated that Diphoterine was able to minimise pain and expedite wound healing by increasing the level of beta-endorphin and inhibiting the release of substance P.^13^ In addition, a few clinical studies have highlighted that Diphoterine could reduce the severity of CEI when compared to normal saline irrigation.^10,11^ Although the use of Diphoterine has been reported and adopted in certain parts of Europe,^10,11,14^ only one case has been reported in the United Kingdom in relation to the efficacy of Diphoterine for ocular and cutaneous burn.^9^

To the best of our knowledge, this study represents the first case series in the United Kingdom examining the clinical outcomes and safety of Diphoterine irrigation for CEI. The effectiveness of Diphoterine was demonstrated by the quick neutralisation of the pH, despite being given in a significantly smaller volume compared to normal saline. The much smaller volume of irrigation fluid needed to neutralise pH has been supported in other studies, reporting up to 17 times less volume with Diphoterine compared to water for neutralisation.^15^ In the setting of CEI when the patients are often in pain and distressed, using an irrigation fluid which is effective and requires less volume is beneficial for the patients, particularly for children who are usually not tolerant to irrigation. In our experience, Diphoterine was well tolerated by all our patients, including the 3-year-old child, and we did not observe any adverse events in the majority of patients, except for patient 3 who presented with a grade-IV CEI. In addition, rapid neutralisation of the pH helps reduce the irrigation time and improve workflow in various clinical care settings, including the community, emergency department, and eye casualty.

The three cases presented above highlight the utility of Diphoterine in various settings. Case 1 (Roper-Hall grade 1) shows the advantage of using Diphoterine for patient with refractory pH, with a relatively smaller volume of fluid needed for Diphoterine than normal saline. Case 2 demonstrated the advantage of using smaller volume of irrigation fluid such as Diphoterine in effectively managing CEI in the paediatric population. Case 3 features how a persistently high pH of 8 (despite 3 L of saline irrigation) was lowered to 7.5 with only 200 mL of Diphoterine. Although the patient presented within 30 mins of the injury, the severity of the initial damage had led to a near-total LSCD. It would be valuable to examine whether the choice of initial first-aid irrigation (Diphoterine vs normal saline) would have changed the prognosis but this could only be determined in larger case–control studies.

It is noteworthy to mention that the effectiveness of the irrigation fluids used in this study is based on their ability in neutralising the surface pH, which may not reflect the aqueous pH. Previous ex vivo experimental studies of alkali CEI have shown that aqueous pH may continue to rise and stay elevated despite prolonged rinsing of the ocular surface, suggesting that the initial normalisation of the surface pH (without the aqueous pH measurement) may provide a false reassurance.^16,17^ However, surface pH measurement is a standard practice that is widely adopted in many countries, including the United Kingdom, due to its non-invasive nature and technical simplicity, as opposed to aqueous humour pH measurement (via a paracentesis), which is an invasive procedure with risk of lens damage and endophthalmitis. In addition, it may not be practical as a part of the routine assessment in the general emergency departments who are not staffed with ophthalmologists. Based on the surface pH measurement, we were able to demonstrate that Diphoterine could achieve a faster neutralisation of the surface pH than normal saline, with the advantage of requiring a much smaller volume of irrigation, highlighting its beneficial role in the real-world setting.

The diffusion characteristic of the irrigation fluids serves as another important aspect that guides the choice of the fluid. In vitro and ex vivo studies have shown that normal saline was much less effective than Diphoterine and water in normalising the aqueous pH.^16,17^ The mechanism underlying this can be explained by the differences in osmolarity following CEI. The high osmolarity of the corneal stroma following a chemical burn draws in the hypoosmolaric water resulting in dilution with minimal effect on the pH, with resultant corneal oedema. However, for normal saline (which is isotonic relative to the corneal stroma), there is a push inside of high osmolarities with resultant increase in intraocular pH; therefore, the normalisation of intraocular pH is not as effective, which was shown in the experiments by Rihawi and co-authors.^16,17^ Among all the investigated irrigating fluids, Diphoterine was reported as being the most effective fluid in neutralising intraocular pH by means of hyperosmolar water and OH^−^ ions efflux from the cornea and chemically neutralising the ocular surface. By this, the intraocular and extraocular pH is normalised for alkali and acids.^16^

We also acknowledge that this study was limited by the small sample size in a single tertiary referral centre. Nevertheless, we highlighted that Diphoterine is a safe and effective irrigation fluid in rapidly neutralising the surface pH during the management of CEI. This is particularly relevant in children or uncooperative patients with severe CEI that requires rapid pH neutralisation to limit tissue damage. The effectiveness, safety profile, ease of administration, and tolerability of Diphoterine irrigation observed in our study warrant further exploration and consideration for routine adoption in the community and clinical practice.

## References

[1] Dua HS Ting DSJ Al Saadi A , et al. Chemical eye injury: pathophysiology, assessment and management. Eye 2020; 34: 2001–2019. 32572184 10.1038/s41433-020-1026-6 PMC7784957

[2] Ghosh S Salvador-Culla B Kotagiri A , et al. Acute chemical eye injury and limbal stem cell deficiency – a prospective study in the United Kingdom. Cornea 2019; 38: 8–12. 30199398 10.1097/ICO.0000000000001739

[3] Bizrah M Yusuf A Ahmad S . An update on chemical eye burns. Eye 2019; 33: 1362–1377. 31086244 10.1038/s41433-019-0456-5 PMC7002428

[4] Ahmmed AA Ting DSJ Figueiredo FC . Epidemiology, economic and humanistic burdens of ocular surface chemical injury: a narrative review. Ocul Surf 2021; 20: 199–211. 33647471 10.1016/j.jtos.2021.02.006

[5] Scott WJ Schrage N Dohlman C . Emergency eye rinse for chemical injuries: new considerations. JAMA Ophthalmol 2015; 133: 245. 25474079 10.1001/jamaophthalmol.2014.5045

[6] Alexander KS Wasiak J Cleland H . Chemical burns: Diphoterine untangled. Burns 2018; 44: 752–766. 29029860 10.1016/j.burns.2017.09.017

[7] Fortin JL Fontaine M Bodson L , et al. Use of an amphoteric solution in eye, skin and oral chemical exposures: retrospective multicenter clinical case series. J Clin Toxicol 2017; 7: 343.

[8] Hall AH Cavallini M Mathieu L , et al. Safety of dermal Diphoterine application: an active decontamination solution for chemical splash injuries. Cutan Ocul Toxicol 2009; 28: 149–156. 19888884 10.3109/15569520903269122

[9] Lewis CJ Al-Mousawi A Jha A , et al. Is it time for a change in the approach to chemical burns? The role of Diphoterine(®) in the management of cutaneous and ocular chemical injuries. J Plast Reconstr Aesthet Surg 2017; 70: 563–567. 28330646 10.1016/j.bjps.2017.02.013

[10] Merle H Donnio A Ayeboua L , et al. Alkali ocular burns in Martinique (French West Indies) Evaluation of the use of an amphoteric solution as the rinsing product. Burns 2005; 31: 205–211. 15683694 10.1016/j.burns.2004.09.001

[11] Wiesner N Dutescu RM Uthoff D , et al. First aid therapy for corrosive chemical eye burns: results of a 30-year longitudinal study with two different decontamination concepts. Graefes Arch Clin Exp Ophthalmol 2019; 257: 1795–1803. 31147840 10.1007/s00417-019-04350-x

[12] Roper-Hall MJ . Thermal and chemical burns. Trans Ophthalmol Soc U K 1965; 85: 631–653. 5227208

[13] Hall AH Blomet J Mathieu L . Diphoterine for emergent eye/skin chemical splash decontamination: a review. Vet Hum Toxicol 2002; 44: 228–231. 12136973

[14] Lynn DD Zukin LM Dellavalle R . The safety and efficacy of Diphoterine for ocular and cutaneous burns in humans. Cutan Ocul Toxicol 2017; 36: 185–192. 27486965 10.1080/15569527.2016.1217423

[15] Fosse C Mathieu L Hall AH , et al. Decontamination of tetramethylammonium hydroxide (TMAH) splashes: promising results with Diphoterine in vitro. Cutan Ocul Toxicol 2010; 29: 110–115. 20233016 10.3109/15569521003661288

[16] Rihawi S Frentz M Schrage NF . Emergency treatment of eye burns: which rinsing solution should we choose? Graefes Arch Clin Exp Ophthalmol 2006; 244: 845–854. 16365735 10.1007/s00417-005-0034-3

[17] Rihawi S Frentz M Becker J , et al. The consequences of delayed intervention when treating chemical eye burns. Graefes Arch Clin Exp Ophthalmol 2007; 245: 1507–1513. 17492301 10.1007/s00417-007-0597-2

